# Expression of Antimicrobic Peptide Piscidin1 in Gills Mast Cells of Giant Mudskipper *Periophthalmodon schlosseri* (Pallas, 1770)

**DOI:** 10.3390/ijms232213707

**Published:** 2022-11-08

**Authors:** Alessio Alesci, Gioele Capillo, Doaa M. Mokhtar, Angelo Fumia, Roberta D’Angelo, Patrizia Lo Cascio, Marco Albano, Maria Cristina Guerrera, Ramy K. A. Sayed, Nunziacarla Spanò, Simona Pergolizzi, Eugenia Rita Lauriano

**Affiliations:** 1 Department of Chemical, Biological, Pharmaceutical and Environmental Sciences, University of Messina, 98166 Messina, Italy; 2 Department of Biomedical, Dental and Morphological and Functional Imaging, University of Messina, 98125 Messina, Italy; 3 Institute of Marine Biological Resources and Biotechnology, National Research Council (IRBIM, CNR), 98164 Messina, Italy; 4 Department of Anatomy and Histology, Faculty of Veterinary Medicine, Assiut University, Assiut 71526, Egypt; 5 Department of Clinical and Experimental Medicine, University of Messina, Padiglione C, AOU Policlinico ‘G. Martino’, 98124 Messina, Italy; 6 Department of Veterinary Sciences, University of Messina, 98168 Messina, Italy; 7 Department of Anatomy and Embryology, Faculty of Veterinary Medicine, Sohag University, Sohag 82524, Egypt

**Keywords:** immune system, gills, amphibious teleost, confocal microscopy, Piscidin1

## Abstract

The amphibious teleost Giant mudskipper (*Periophthalmodon schlosseri*, Pallas 1770) inhabit muddy plains and Asian mangrove forests. It spends more than 90% of its life outside of the water, using its skin, gills, and buccal-pharyngeal cavity mucosa to breathe in oxygen from the surrounding air. All vertebrates have been found to have mast cells (MCs), which are part of the innate immune system. These cells are mostly found in the mucous membranes of the organs that come in contact with the outside environment. According to their morphology, MCs have distinctive cytoplasmic granules that are released during the degranulation process. Additionally, these cells have antimicrobial peptides (AMPs) that fight a variety of infections. Piscidins, hepcidins, defensins, cathelicidins, and histonic peptides are examples of fish AMPs. Confocal microscopy was used in this study to assess Piscidin1 expression in Giant Mudskipper branchial MCs. Our results demonstrated the presence of MCs in the gills is highly positive for Piscidin1. Additionally, colocalized MCs labeled with TLR2/5-HT and Piscidin1/5-HT supported our data. The expression of Piscidin1 in giant mudskipper MCs highlights the involvement of this peptide in the orchestration of teleost immunity, advancing the knowledge of the defense system of this fish.

## 1. Introduction

The giant mudskipper, *Periophthalmodon schlosseri* (Pallas, 1770), is an amphibious and air-breathing euryhaline teleost. This species lives in close association with mangrove forests and spends more than 90% of its time out of the water in adjacent mud plains, therefore it has evolved morphological and physiological adaptations to suit its unique life habit [1,2]. The changes in the gill structure and the hypervascularization of the epithelial tissues, facilitate air-breathing during out-of-water life. The respiratory gas exchange occurs through the gills and the upper vascularized epithelium covering the cavities in these fishes, which either take the air directly through the skin or hold it in their large oropharyngeal-opercular cavities [3].

Structural modifications that assist air respiration, probably present in all mudskippers, have been also identified in giant mudskipper [4,5]. They possess an inhalant aperture in the gills and operculum, a well-vascularized gill membrane covering the gills, and an augmentation in the volume of the opercular and pharyngeal chambers equal to about 1/6 of the body volume of this fish [6]. Mudskippers are highly developed teleosts that have evolved for an amphibious lifestyle in mud plains, therefore their terrestrial activity is connected to the ability to breathe air, exactly like amphibians [3].

Mast cells (MCs) are ovoid or rounded immune cells with amoeboid activity that are found in a wide range of organs and peripheral tissues, including the skin, blood vessels, digestive tract, and gills [7]. These cells are also involved in several defense mechanisms of the body, being able to modulate inflammation [8]. According to their morphology, MCs include distinctive cytoplasmic granules that store bioactive mediators including histamine and serotonin [9]. These cells have been characterized in all classes of vertebrates [10]. MCs in teleosts have functional traits that are very comparable to those of mammals, according to numerous studies. Additionally, the MCs of fish include granules that share components with mammalian granules, such as lysozyme, serotonin, and specific antimicrobial peptides (AMPs) called Piscidins [11].

AMPs, a family of low molecular weight peptides and proteins, are present in all forms of life, from prokaryotes to eukaryotic plants and animals [12]. In low vertebrates, these peptides play a crucial role in the innate immune system [13]. Piscidins are AMPs composed of 22 alpha-helix residues that were originally found in fish gills and skin [3,14]. They play a protective role against pathogens and foreign agents. There is evidence that Piscidins are present in a wide range of teleosts, including the families Moronidae, Sciaenidae, Siganidae, Belontidae, Cichlidae, Percichthyidae, Latidae Dae Sparidae, Sygnathidae, and Latridae. They were originally isolated from the striped bass (*Morone saxatilis*, Walbaum 1792), the white bass (*Morone chrysops*, Rafinesque 1820), and their hybrid [15]. Genetic transcriptions of Piscidin have been characterized in striped bass and white bass [16], European seabass (*Dicentrarchus labrax*, Linnaeus 1758) [17], Nile tilapia (*Oreochromis niloticus*, Linnaeus 1758) [18], Atlantic cod (*Gadus morhua*, Linnaeus 1758) [19], and goldfish (*Carassius auratus*, Linnaeus 1758) [20]. Piscidin exhibits potent broad-spectrum activity against bacteria and parasites [21] and has been localized in MCs [22,23,24]. Therefore, these cells play a key role in teleost inflammatory response mechanisms [25,26].

This study aims to characterize for the first time MCs in the gills of giant mudskipper with anti-Piscidin1 antibodies, to provide further knowledge about the immune system of this teleost which shows peculiar characteristics common both to fish and amphibians.

## 2. Results

**Figure 1:**
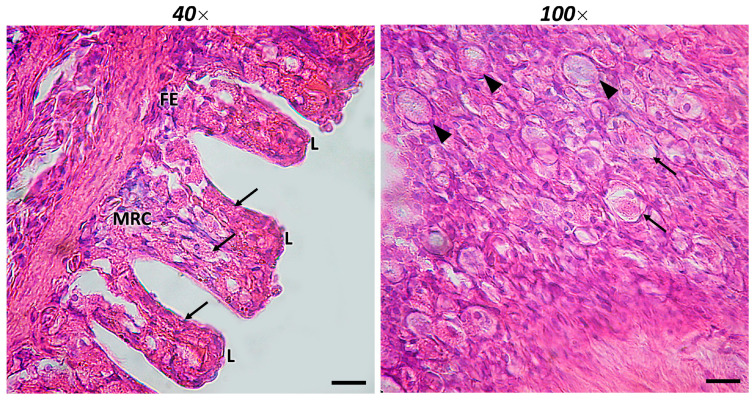
Section of the gill of giant mudskipper stained with H/E. In 40× (scale bar 40 μm) the presence of filaments (FE) and lamellar epithelium (L) can be noted. Mitochondria-rich cells (MRCs) are present in the gill lamellar epithelium. Scattered MCs are also noted (arrows). In 100× (scale bar 100 μm) rodlet cells (RCs) (arrowheads) and MCs can be distinguished.

Giant mudskipper gills consist of gill arches, secondary lamellae, primary lamellae, and arteries. As in teleosts, it has four pairs of gill arches divided into two parallel hemibranchs. On both surfaces of the gill filaments, there are alternate sets of lamellae. These interdigitating filaments, similar to those found in a majority of respiratory fishes, have tiny interbranchial septa. Squamous and columnar pavement cells, large mucous cells, and mitochondrial-rich cells (MRCs) cover the gill filaments and lamellae. Scattered MCs are observed in the secondary and primary lamellae, and the pharyngeal portion of the gills (Figure 1).

**Figure 2:**
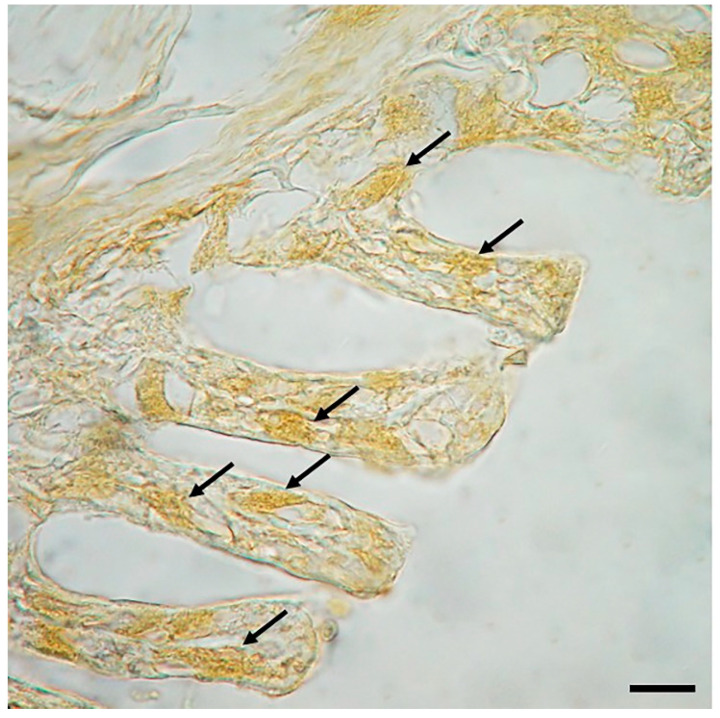
Section of the gill of giant mudskipper, immunoperoxidase, 40×, scale bar 40 μm. MCs positive to Piscidin1 can be seen in the lamellae (arrows).

Anti-Piscidin1 immunoperoxidase shows clear and well-defined reactive MCs to the antibody tested, as evidenced by the DAB reaction (Figure 2).

**Figure 3:**
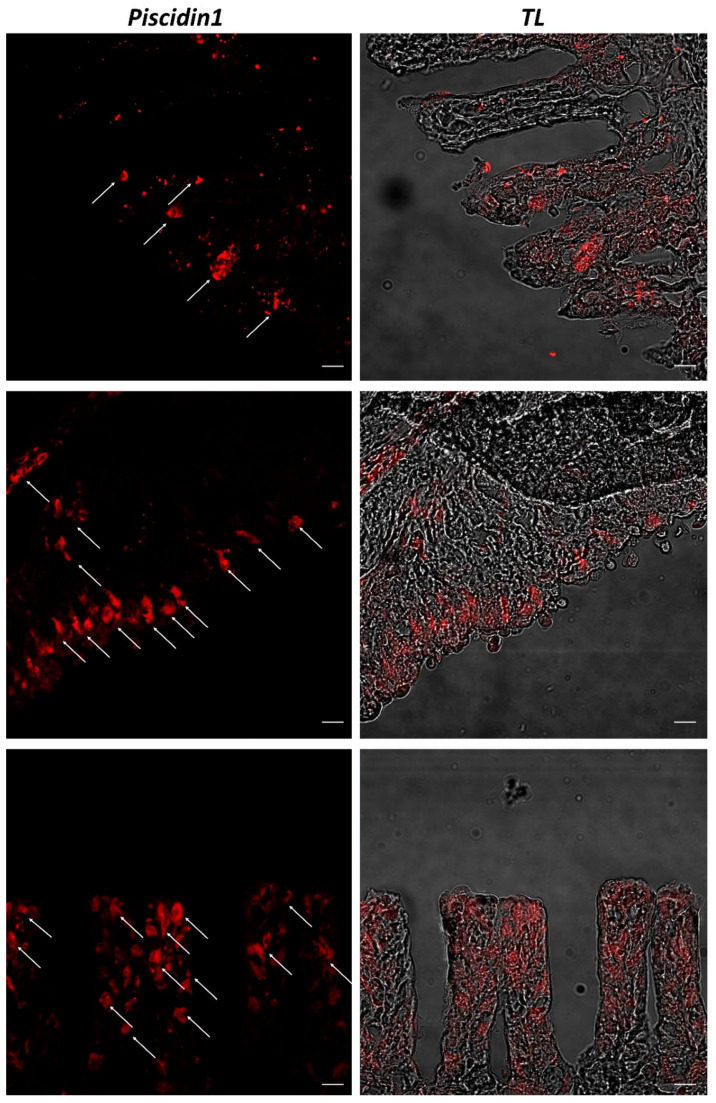
Section of the gill of giant mudskipper, immunofluorescence, 20×, scale bar 20 μm. MCs immunoreactive to Piscidin1 can be noted (arrows). TL = transmitted light.

Confocal microscopy shows MCs well localized in lamellar tissue and gill pharyngeal portion, immunopositive to Piscidin1 antibody, as also shown by transmitted light images (Figure 3).

**Figure 4:**
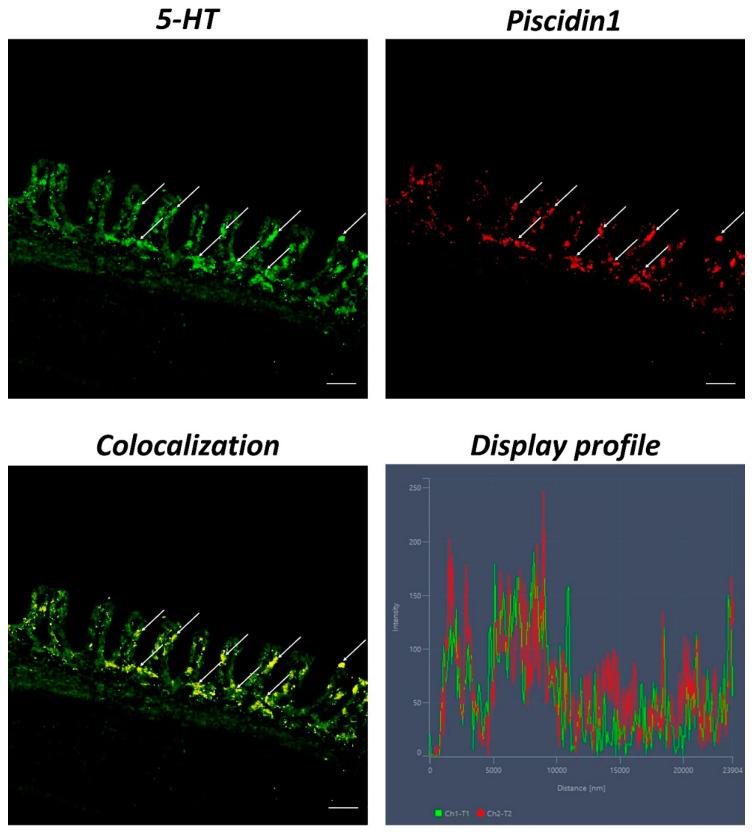
Section of the gill of giant mudskipper, immunofluorescence of 5-HT and Piscidin1, 40×, scale bar 40 μm. MCs positivity to 5-HT (green) and Piscidin1 (red) can be clearly seen (arrows). The colocalization of the antibodies is confirmed by the ‘display profile’ function.

An evident colocalization between Piscidin1 and 5-HT can be noted in MCs. The use of the “Display profile” function of the confocal microscope supported our data, highlighting the fluorescence peaks at the individual localization and the colocalization, consistent with the results obtained (Figure 4).

**Figure 5:**
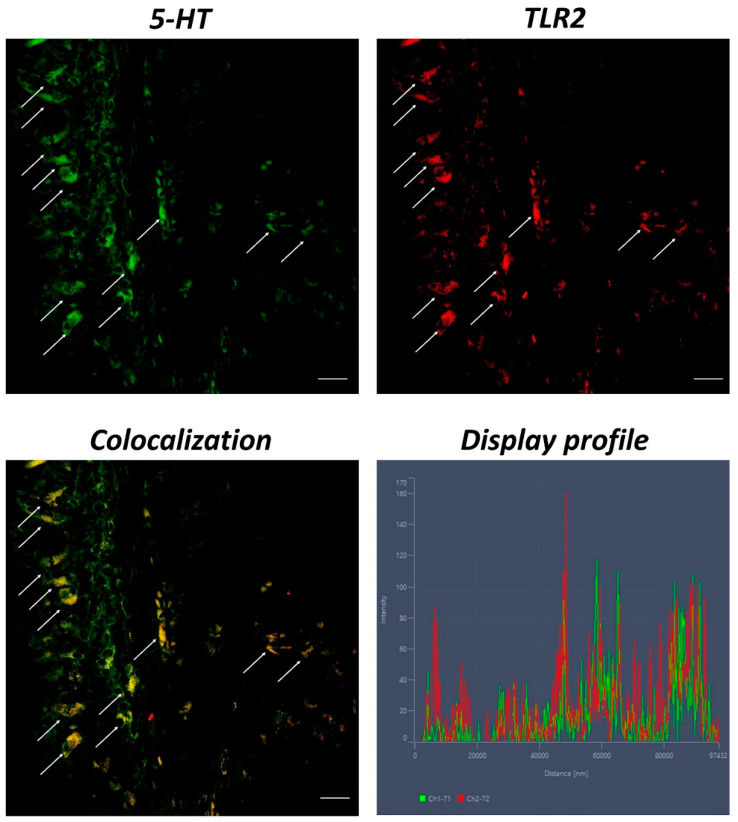
Section of the gill of giant mudskipper, immunofluorescence of 5-HT and TLR2, 40×, scale bar 40 μm. Scattered MCs positive to 5-HT (green) and TLR2 (red) are evident (arrows). The colocalization of the antibodies is clear and confirmed by the ‘display profile’ function.

Anti-TLR2 and anti-5-HT labeled immunoreactive and localized MCs. The “Display profile” function confirmed our data (Figure 5).

Quantitative analysis revealed an equivalent number of MCs detected with the antibodies tested (Table 1).

## 3. Discussion

AMPs are a group of short peptides in vertebrates for innate immunity to fight exogenous pathogens [27]. Piscidins, histonic peptides, hepcidins, cathelicidins, and defensins are fish AMPs. Piscidin is powerful and broad-spectrum, highly conserved among the Acanthopterygii superorder [28]. Piscidin, pleurocidin, epinecidin, moronecidin, gaduscidin, crisophsin, misgurin, dicentracin, and mixinidine are members of the piscidin family. Piscidin is a linear α-helic cationic peptide [29] that has been characterized in various species of teleost [30]. It has antibacterial, antifungal, and antiviral properties. Innate immune defense against parasitic infections was also demonstrated by this peptide [31,32]. Several isoforms of Piscidin have been characterized, such as Piscidin1 and Piscidin2 from *Epinephelus malabaricus* (Bloch and Schneider, 1804) [33]. Salger et al. (2016) identified nine isoforms of piscidin in striped bass and white bass and verified the expression of eight orthologous forms in the hybrid striped bass. At least, six different piscidin loci exist: four loci possess two alleles (piscidin 1, 3, 4, and 6), and two loci possess one allele (piscidin 5 and 7) [16]. A study by Ruangsri et al. (2012) shows the ubiquitous presence of Piscidin1 in the organs of Atlantic cod. Its presence in immune-related tissues raises the possibility that this peptide plays a significant role in the innate immune system of cod. Furthermore, the localization of Piscidin1 in some non-immune tissues and organs, such as the kidney, gut, respiratory epithelial cells, the swim bladder, the skin, the liver, the heart, the eye, and the oocytes, highlights further potential uses for this ubiquitous peptide and its capacity to keep Atlantic cod homeostasis [34]. Piscidin1 is mainly found in the gill tissues, muscles, cranial kidneys, skin, and intestine of the teleosts [28]. Many species possess AMPs with strong biological functions, demonstrating their ubiquitous occurrence and the critical roles they play as the first line of host defense [35].

Mudskippers are a typical group of fish species, largely studied for the transition from aquatic to terrestrial lifestyles. In comparison to other fish, it has been found that mudskippers have overexpression of innate immune system genes that may act as a defense against terrestrial pathogens. Positive selection has probably promoted several ammonia excretion pathway genes in the gills, indicating their critical involvement in mudskippers’ ability to withstand environmental ammonia [36]. The gills of the mudskipper have developed a morpho-functional and structural adaptation due to the environmental conditions in which the fish lives. They are relatively small compared to the gills of other fishes and have a larger opercular space to receive incoming water during the breathing process [5]. In addition, gills, skin, and mucous membranes are more exposed to the external environment and are equipped with associated lymphoid tissues (gills-associated lymphoid tissue GIALT, skin-associated lymphoid tissue SALT, mucosa-associated lymphoid tissue MALT, gut-associated lymphoid tissue GALT, etc.) that provide them with a plethora of immune cells such as MCs, rodlet cells, macrophages, granulocytes, lymphocytes, dendritic cells, involved in the protection of the organism [37,38].

MCs travel into the blood from peripheral tissues, colonizing them, thus differentiating in response to microenvironmental stimuli [39]. Comparative investigations have reported granule cells in all vertebrate classes that share the main characteristics of MCs [40]. These cells contain metachromatic granules in their cytoplasm that store a plethora of secretory molecules, including histamine and serotonin. Tryptase and serotonin, two related substances, have been found in the MCs of teleost fishes [41]. More evolved fish species exhibit a population of granular cells with general properties typical of the MCs of superior vertebrates. Considering that these cells are crucial to the immunological response, the MCs phenotype is recognized in all vertebrate classes, indicating their evolutionary conservation [42]. Due to their susceptibility to external infections, MCs are concentrated in the skin, intestines, gills, and respiratory system [20,38].

In this study, we have characterized for the first time MCs in the gills of giant mudskipper with Piscidin1. MCs immunoreactive to piscidin are most common at pathogen entry sites, including skin, gills, and gastrointestinal tract [42]. In a previous study, we demonstrated the expression of Piscidin in intestinal MCs of *Carassius auratus* [10]. Dezfuli et al. (2010) demonstrated that MCs in the gills of European seabass expressed piscidin under physiological and pathological conditions [31]. Following these data, our results show MCs positive for Piscidin1 in the gills of giant mudskipper labeled with the immunoperoxidase method. In addition, to further validate our data, MCs were labeled with Piscidin1 with immunofluorescence by using a confocal microscope, noting a marked immunoreactivity of these cells to the antibody tested. To further confirm our results on the expression of Piscidin1 in MCs, these immune cells were also colocalized with anti-5-HT/anti-Piscidin1 and anti-5-HT/anti-TLR2 antibodies.

MCs express a range of pattern-recognition receptors, including TLRs [43]. It is known that TLRs are highly phylogenetically conserved across all vertebrate classes [44,45,46,47,48], and involved in immune response [49,50]. Several studies have characterized TLR2 in immune cells in different tissues and organs of fish and ascidians [1,51,52]. MCs are among the earliest inflammatory cells with the ability to combat infectious microorganisms and initiate immune responses via TLRs [53]. Serotonin, a tryptamine synthesized from the amino acid tryptophan (5-HTP), is an important neurotransmitter and trophic factor, and it is synthesized and stored by both MCs and neurons [54]. It represents a link between the immune and endocrine systems [55] and is highly conserved among vertebrates [3]. In fish, it is stored in considerable quantities by MCs and helps to orchestrate the immune response [56,57,58]. In our previous study (2022), we demonstrated the presence of TLR2 and 5-HT in MCs of the intestine of *Carassius auratus* [10]. Following these researches, we found immunoreactive colocalized MCs to Piscidin1 and 5-HT, and TLR2 and 5-HT in giant mudskipper gills. The “Display profile” function corroborated these results, highlighting the fluorescence peaks expressed by the colocalization of all antibodies.

## 4. Materials and Methods

### 4.1. Samples

The preparations for optical and confocal microscopy as well as paraffin block storage were made using samples of giant mudskipper gills that were taken from the histotheca of our laboratory.

### 4.2. Tissue Preparation

The samples were fixed in 4% paraformaldehyde in 0.1 M phosphate-buffered saline (pH 7.4), rinsed in xylene, and embedded in Paraplast^®^ after being dehydrated in graded ethanol for 12 to 18 h (McCormick Scientific LLC, St. Louis, MO, USA). Lastly, serial slices (3–5 μm thick) were cut using a rotary microtome (LEICA 2065 Supercut, Nussloch, Germany, Europe) [59].

### 4.3. Histology

Serial slices were stained for light microscopic examination using Hematoxylin-Eosin (H/E) (05-B06008/A + 05-M10002 BioOptica Milano S.p.A, Milan, Italy, Europe) [60].

### 4.4. Immunoperoxidase

Piscidin1 was examined with an optical microscope and immunohistochemical techniques. Slices were treated with an anti-Piscidin1 antibody overnight in a humid environment. Following a PBS wash, the sections were incubated with a goat anti-rabbit IgG-peroxidase conjugate for 60 min (Sigma-Aldrich, St. Louis, MO, USA, dilution 1:100, source Goat). By allowing the sections to sit in a solution of 0.02% diaminobenzidine (DAB) and 0.015% hydrogen peroxide for 1–5 min at room temperature, the peroxidase activity of the sections was assessed. Sections were dehydrated, mounted, and inspected with a Zeiss Axioskop 2 plus microscope and a Sony Digital Camera DSC-85 after being rinsed in PBS. As a negative control, experiments were conducted without the primary antibody.

### 4.5. Immunofluorescence

Serial slices were treated with 2.5% bovine serum albumin (BSA) for an hour before being gradually deparaffinized, rehydrated in PBS, and blocked. The sections were treated with primary antibodies against Piscidin1, Serotonin (5-HT), and Toll-like Receptor 2 (TLR2) in a humid chamber overnight at 4 °C [61]. The sections were then evaluated individually and in double-label tests. We used secondary antibodies from Molecular Probes, Invitrogen, Eugene, OR, USA, 1:300, Alexa Fluor 594 donkey anti-rabbit IgG TRITC conjugated, and Alexa Fluor 488 donkey anti-mouse IgG FITC conjugated. To prevent photobleaching, the sections were mounted with Vectashield (Vector Labs, Burlingame, CA, USA), and the cover was removed after washing. As a negative control, experiments were conducted without the primary antibodies. Rat skin tissues were used as a positive control to verify the immunopositivity of the primary antibodies. Table 2 summarizes the information on antibodies.

### 4.6. Laser Confocal Immunofluorescence

Sections were examined, and photographs were acquired using a Zeiss LSM DUO confocal laser scanning microscope with a META module (Carl Zeiss MicroImaging GmbH, Jena, Germany, Europe). This microscope has two helium-neon lasers (543 and 633 l) as well as two argon lasers (458 and 488 l). By digitizing each image, a 2048-by-2048 pixel array with an 8-bit resolution was produced. Optical slices of fluorescence samples were produced using helium-neon (543 nm) and argon (458 nm) lasers with scanning speeds of 1 min and 2 s and up to eight averages. Zen 2011 was used to enhance the photographs (LSM 700 Zeiss software, Oberkochen, Germany, Europe). To avoid photo deterioration, each picture was snapped immediately. To create the figure montage, digital photo cropping was done in Adobe Photoshop CC (Adobe Systems, San Jose, CA, USA). The intensity profile of an image was rendered on a freely selected line using the “display profile” feature of the laser scanning microscope. Graphs showing the intensity curves and scanned images are displayed together.

### 4.7. Quantitative Analysis

For each sample, 5 sections and 10 fields were evaluated to collect data for the quantitative analysis.

The observation fields were chosen based on how much the cells responded. Using ImageJ software, each field was evaluated. The background was removed, the image was converted to 8 bits, and the cells were identified using a “Threshold” filter and a mask. The “Analyze particles” plug-in was then utilized to count the cells. SigmaPlot version 14.0 (Systat Software, San Jose, CA, USA) was used to count the number of MCs that were positive for Piscidin1, 5-HT, and TLR2 in each field. The normally distributed data were analyzed using One-way ANOVA, then Student’s t-test. The mean values and standard deviations (SD) of the data are displayed. Statistical significance was given to the following *p* values in this order: ** *p* ≤ 0.01, * *p* ≤ 0.05.

## 5. Conclusions

In conclusion, our results show for the first time the expression of Piscidin1 in MCs in the gills of giant mudskipper, suggesting phylogenetic conservation of these cells even in a fish so particular that it can be considered as an evolutionary link between fishes and amphibians. Moreover, we cannot exclude that our anti-Piscidin1 antiserum may also recognize other isoforms of piscidin. Further studies could be necessary to support our results. However, our data may provide additional insights into the role of Piscidin1 in the evolution of the immune system in vertebrates.

## Figures and Tables

**Table 1:** Quantitative analysis results (mean values ± standard deviations; *n* = 3).

	No. of MCs
Piscidin1 (immunoperoxidase)	148.37 ± 12.76 **
Piscidin1 (immunofluorescence)	154.43 ± 14.69 *
TLR2	147.39 ± 13.56 **
5-HT	145.92 ± 17.84 *
Piscidin1 + 5-HT	143.68 ± 21.11 *
TLR2 + 5-HT	139.58 ± 16.14 **

** *p* ≤ 0.01. * *p* ≤ 0.05.

**Table 2:** Antibodies data.

Antibody	Supplier	Dilution	Animal Source
Piscidin1	GenScript Biotech Corporation, Rijswijk, Netherlands, Europe. Produced on demand	1:50	Rabbit
TLR2	Active Motif, La Hulpe, Belgium, Europe.	1:125	Rabbit
5-HT	Santa Cruz Biotechnology, Inc., Dallas, TX, USA.	1:50	Mouse
Goat anti-Rabbit IgG Peroxidase conjugated	Sigma Aldrich, Saint Louis, MO, USA.	1:100	Goat
Alexa Fluor 488 Donkey anti-Mouse IgG FITC conjugated	Molecular Probes, Invitrogen	1:300	Donkey
Alexa Fluor 594 Donkey anti-Rabbit IgG TRITC conjugated	Molecular Probes, Invitrogen	1:300	Donkey

## Data Availability

Not applicable.

## References

[1] Lauriano E.R., Faggio C., Capillo G., Spanò N., Kuciel M., Aragona M., Pergolizzi S. (2018). Immunohistochemical Characterization of Epidermal Dendritic-like Cells in Giant Mudskipper, Periophthalmodon Schlosseri. Fish Shellfish Immunol..

[2] Kuciel M., Rita Lauriano E., Silvestri G., Żuwała K., Pergolizzi S., Zaccone D. (2014). The Structural Organization and Immunohistochemistry of G-Protein Alpha Subunits in the Olfactory System of the Air-Breathing Mudskipper, Periophthalmus Barbarus (Linnaeus, 1766) (Gobiidae, Oxudercinae). Acta Histochem..

[3] Zaccone G., Lauriano E.R., Kuciel M., Capillo G., Pergolizzi S., Alesci A., Ishimatsu A., Ip Y.K., Icardo J.M. (2017). Identification and Distribution of Neuronal Nitric Oxide Synthase and Neurochemical Markers in the Neuroepithelial Cells of the Gill and the Skin in the Giant Mudskipper, Periophthalmodon Schlosseri. Zoology.

[4] Yadav A.N., Prasad M.S., Singh B.R. (1990). Gross Structure of the Respiratory Organs and Dimensions of the Gill in the Mud–Skipper, Periophthalmodon Schlosseri (Bleeker). J. Fish Biol..

[5] Supriyati H., Apriliani N.S., Luthfi M.J. (2019). Histological Study of Mudskipper (Periophthalmus Gracilis) Gills. Proceed. Int. Conf. Sci. Eng..

[6] Aguilar N.M., Ishimatsu A., Ogawa K., Huat K.K. (2000). Aerial Ventilatory Responses of the Mudskipper, Periophthalmodon Schlosseri, to Altered Aerial and Aquatic Respiratory Gas Concentrations. Comp. Biochem. Physiol. A Mol. Integr. Physiol..

[7] Galli S.J., Nakae S., Tsai M. (2005). Mast Cells in the Development of Adaptive Immune Responses. Nat. Immunol..

[8] Abraham S.N., St. John A.L. (2010). Mast Cell-Orchestrated Immunity to Pathogens. Nat. Rev. Immunol..

[9] Shim J.K., Caron M.A., Weatherly L.M., Gerchman L.B., Sangroula S., Hattab S., Baez A.Y., Briana T.J., Gosse J.A. (2019). Antimicrobial Agent Triclosan Suppresses Mast Cell Signaling via Phospholipase D Inhibition. J. Appl. Toxicol..

[10] Alesci A., Pergolizzi S., Fumia A., Calabrò C., Lo Cascio P., Lauriano E.R. (2022). Mast Cells in Goldfish (*Carassius Auratus*) Gut: Immunohistochemical Characterization. Acta Zool..

[11] Mulero I., Sepulcre M.P., Meseguer J., García-Ayala A., Mulero V. (2007). Histamine Is Stored in Mast Cells of Most Evolutionarily Advanced Fish and Regulates the Fish Inflammatory Response. Proc. Natl. Acad. Sci. USA.

[12] Augustyniak D., Nowak J., Lundy F.T. (2012). Direct and Indirect Antimicrobial Activities of Neuropeptides and Their Therapeutic Potential. Curr. Protein Pept. Sci..

[13] Zasloff M. (2002). Antimicrobial Peptides of Multicellular Organisms. Nature.

[14] Zaccone G., Capillo G., Fernandes J.M.O., Kiron V., Lauriano E.R., Alesci A., Cascio P.L., Guerrera M.C., Kuciel M., Zuwala K. (2022). Expression of the Antimicrobial Peptide Piscidin 1 and Neuropeptides in Fish Gill and Skin: A Potential Participation in Neuro-Immune Interaction. Mar. Drugs.

[15] Diamond G., Beckloff N., Weinberg A., Kisich K. (2009). The Roles of Antimicrobial Peptides in Innate Host Defense. Curr. Pharm. Des..

[16] Salger S.A., Cassady K.R., Reading B.J., Noga E.J. (2016). A Diverse Family of Host-Defense Peptides (Piscidins) Exhibit Specialized Anti-Bacterial and Anti-Protozoal Activities in Fishes. PLoS ONE.

[17] Lauth X., Shike H., Burns J.C., Westerman M.E., Ostland V.E., Carlberg J.M., Van Olst J.C., Nizet V., Taylor S.W., Shimizu C. (2002). Discovery and Characterization of Two Isoforms of Moronecidin, a Novel Antimicrobial Peptide from Hybrid Striped Bass. J. Biol. Chem..

[18] Salerno G., Parrinello N., Roch P., Cammarata M. (2007). CDNA Sequence and Tissue Expression of an Antimicrobial Peptide, Dicentracin; a New Component of the Moronecidin Family Isolated from Head Kidney Leukocytes of Sea Bass, Dicentrarchus Labrax. Comp. Biochem. Physiol. B Biochem. Mol. Biol..

[19] Acosta J., Montero V., Carpio Y., Velázquez J., Garay H.E., Reyes O., Cabrales A., Masforrol Y., Morales A., Estrada M.P. (2013). Cloning and Functional Characterization of Three Novel Antimicrobial Peptides from Tilapia (Oreochromis Niloticus). Aquaculture.

[20] Alesci A., Pergolizzi S., Capillo G., Cascio P.L., Lauriano E.R. (2022). Rodlet Cells in Kidney of Goldfish (Carassius Auratus, Linnaeus 1758): A Light and Confocal Microscopy Study. Acta Histochem..

[21] Silphaduang U., Noga E.J. (2001). Peptide Antibiotics in Mast Cells of Fish. Nature.

[22] Kim S.Y., Zhang F., Gong W., Chen K., Xia K., Liu F., Gross R., Wang J.M., Linhardt R.J., Cotten M.L. (2018). Copper Regulates the Interactions of Antimicrobial Piscidin Peptides from Fish Mast Cells with Formyl Peptide Receptors and Heparin. J. Biol. Chem..

[23] Silphaduang U., Colorni A., Noga E. (2006). Evidence for Widespread Distribution of Piscidin Antimicrobial Peptides in Teleost Fish. Dis. Aquat. Organ..

[24] Maina J.W., Pringle J.M., Razal J.M., Nunes S., Vega L., Gallucci F., Dumée L.F. (2021). Strategies for Integrated Capture and Conversion of CO_2_ from Dilute Flue Gases and the Atmosphere. ChemSusChem.

[25] Sfacteria A., Brines M., Blank U. (2015). The Mast Cell Plays a Central Role in the Immune System of Teleost Fish. Mol. Immunol..

[26] Romano L.A., Oliveira F.P.S., Pedrosa V.F. (2021). Mast Cells and Eosinophilic Granule Cells in Oncorhynchus Mykiss: Are They Similar or Different?. Fish Shellfish Immunol. Rep..

[27] Chen X., Yi Y., Bian C., You X., Shi Q. (2020). Putative Antimicrobial Peptides in Fish: Using Zebrafish as a Representative. Protein Pept. Lett..

[28] Raju S.V., Sarkar P., Kumar P., Arockiaraj J. (2021). Piscidin, Fish Antimicrobial Peptide: Structure, Classification, Properties, Mechanism, Gene Regulation and Therapeutical Importance. Int. J. Pept. Res. Ther..

[29] Fernandes J.M., Ruangsri J., Kiron V. (2010). Atlantic Cod Piscidin and Its Diversification through Positive Selection. PLoS ONE.

[30] Elumalai P., Rubeena A.S., Arockiaraj J., Wongpanya R., Cammarata M., Ringø E., Vaseeharan B. (2019). The Role of Lectins in Finfish: A Review. Rev. Fish. Sci. Aquac..

[31] Dezfuli B.S., Pironi F., Giari L., Noga E.J. (2010). Immunocytochemical Localization of Piscidin in Mast Cells of Infected Seabass Gill. Fish Shellfish Immunol..

[32] Niu S.-F., Jin Y., Xu X., Qiao Y., Wu Y., Mao Y., Su Y.-Q., Wang J. (2013). Characterization of a Novel Piscidin-like Antimicrobial Peptide from Pseudosciaena Crocea and Its Immune Response to Cryptocaryon Irritans. Fish Shellfish Immunol..

[33] Li Z.-P., Chen D.-W., Pan Y.-Q., Deng L. (2016). Two Isoforms of Piscidin from Malabar Grouper, Epinephelus Malabaricus: Expression and Functional Characterization. Fish Shellfish Immunol..

[34] Ruangsri J., Fernandes J.M.O., Rombout J.H.W.M., Brinchmann M.F., Kiron V. (2012). Ubiquitous Presence of Piscidin-1 in Atlantic Cod as Evidenced by Immunolocalisation. BMC Vet. Res..

[35] Yi Y., You X., Bian C., Chen S., Lv Z., Qiu L., Shi Q. (2017). High-Throughput Identification of Antimicrobial Peptides from Amphibious Mudskippers. Mar. Drugs.

[36] You X., Bian C., Zan Q., Xu X., Liu X., Chen J., Wang J., Qiu Y., Li W., Zhang X. (2014). Mudskipper Genomes Provide Insights into the Terrestrial Adaptation of Amphibious Fishes. Nat. Commun..

[37] Dezfuli B.S., Pironi F., Maynard B., Simoni E., Bosi G. (2022). Rodlet Cells, Fish Immune Cells and a Sentinel of Parasitic Harm in Teleost Organs. Fish Shellfish Immunol..

[38] Alesci A., Cicero N., Fumia A., Petrarca C., Mangifesta R., Nava V., Lo Cascio P., Gangemi S., Di Gioacchino M., Lauriano E.R. (2022). Histological and Chemical Analysis of Heavy Metals in Kidney and Gills of Boops Boops: Melanomacrophages Centers and Rodlet Cells as Environmental Biomarkers. Toxics.

[39] Frossi B., Mion F., Sibilano R., Danelli L., Pucillo C.E.M. (2018). Is It Time for a New Classification of Mast Cells? What Do We Know about Mast Cell Heterogeneity?. Immunol. Rev..

[40] Baccari G.C., Pinelli C., Santillo A., Minucci S., Rastogi R.K. (2011). Mast Cells in Nonmammalian Vertebrates. International Review of Cell and Molecular Biology.

[41] Dobson J.T., Seibert J., Teh E.M., Da’as S., Fraser R.B., Paw B.H., Lin T.-J., Berman J.N. (2008). Carboxypeptidase A5 Identifies a Novel Mast Cell Lineage in the Zebrafish Providing New Insight into Mast Cell Fate Determination. Blood.

[42] Saccheri P., Travan L., Ribatti D., Crivellato E. (2020). Mast Cells, an Evolutionary Approach. Ital. J. Anat. Embryol..

[43] Sandig H., Bulfone-Paus S. (2012). TLR Signaling in Mast Cells: Common and Unique Features. Front. Immunol..

[44] Alesci A., Pergolizzi S., Lo Cascio P., Fumia A., Lauriano E.R. (2021). Neuronal Regeneration: Vertebrates Comparative Overview and New Perspectives for Neurodegenerative Diseases. Acta Zool..

[45] Marino A., Pergolizzi S., Lauriano E.R., Santoro G., Spataro F., Cimino F., Speciale A., Nostro A., Bisignano G. (2015). TLR2 Activation in Corneal Stromal Cells by *Staphylococcus Aureus* -Induced Keratitis. APMIS.

[46] Alesci A., Capillo G., Fumia A., Messina E., Albano M., Aragona M., Lo Cascio P., Spanò N., Pergolizzi S., Lauriano E.R. (2022). Confocal Characterization of Intestinal Dendritic Cells from Myxines to Teleosts. Biology.

[47] Alesci A., Lauriano E.R., Aragona M., Capillo G., Pergolizzi S. (2020). Marking Vertebrates Langerhans Cells, from Fish to Mammals. Acta Histochem..

[48] Alesci A., Pergolizzi S., Savoca S., Fumia A., Mangano A., Albano M., Messina E., Aragona M., Lo Cascio P., Capillo G. (2022). Detecting Intestinal Goblet Cells of the Broadgilled Hagfish Eptatretus Cirrhatus (Forster, 1801): A Confocal Microscopy Evaluation. Biology.

[49] Alesci A., Aragona M., Cicero N., Lauriano E.R. (2021). Can Nutraceuticals Assist Treatment and Improve COVID-19 Symptoms?. Nat. Prod. Res..

[50] Alesci A., Lauriano E.R., Fumia A., Irrera N., Mastrantonio E., Vaccaro M., Gangemi S., Santini A., Cicero N., Pergolizzi S. (2022). Relationship between Immune Cells, Depression, Stress, and Psoriasis: Could the Use of Natural Products Be Helpful?. Molecules.

[51] Alesci A., Pergolizzi S., Lo Cascio P., Capillo G., Lauriano E.R. (2022). Localization of Vasoactive Intestinal Peptide and Toll-like Receptor 2 Immunoreactive Cells in Endostyle of Urochordate *Styela plicata* (Lesueur, 1823). Microsc. Res. Tech..

[52] Alessio A., Pergolizzi S., Gervasi T., Aragona M., Lo Cascio P., Cicero N., Lauriano E.R. (2021). Biological Effect of Astaxanthin on Alcohol-Induced Gut Damage in Carassius Auratus Used as Experimental Model. Nat. Prod. Res..

[53] Metz M., Siebenhaar F., Maurer M. (2008). Mast Cell Functions in the Innate Skin Immune System. Immunobiology.

[54] Nautiyal K.M., Ribeiro A.C., Pfaff D.W., Silver R. (2008). Brain Mast Cells Link the Immune System to Anxiety-like Behavior. Proc. Natl. Acad. Sci. USA.

[55] De Winter B.Y., van den Wijngaard R.M., de Jonge W.J. (2012). Intestinal Mast Cells in Gut Inflammation and Motility Disturbances. Biochim. Biophys. Acta BBA Mol. Basis Dis..

[56] da Silva T.A., Zorzetto-Fernandes A.L.V., Cecílio N.T., Sardinha-Silva A., Fernandes F.F., Roque-Barreira M.C. (2017). CD14 Is Critical for TLR2-Mediated M1 Macrophage Activation Triggered by N-Glycan Recognition. Sci. Rep..

[57] Dezfuli B.S., Lui A., Giovinazzo G., Boldrini P., Giari L. (2009). Intestinal Inflammatory Response of Powan *Coregonus Lavaretus* (Pisces) to the Presence of Acanthocephalan Infections. Parasitology.

[58] Khan N., Deschaux P. (1997). Role of Serotonin in Fish Immunomodulation. J. Exp. Biol..

[59] Zaccone G., Bonga S.E.W., Flik G., Fasulo S., Licata A., Cascio P.L., Mauceri A., Lauriano E.R. (1992). Localization of Calbindin D28K-like Immunoreactivity in Fish Gill: A Light Microscopic and Immunoelectron Histochemical Study. Regul. Pept..

[60] Icardo J.M., Colvee E., Lauriano E.R., Capillo G., Guerrera M.C., Zaccone G. (2015). The Structure of the Gas Bladder of the Spotted Gar, Lepisosteus Oculatus: THE GAS BLADDER OF *Lepisosteus Oculatus*. J. Morphol..

[61] Lauriano E.R., Żuwała K., Kuciel M., Budzik K.A., Capillo G., Alesci A., Pergolizzi S., Dugo G., Zaccone G. (2016). Confocal Immunohistochemistry of the Dermal Glands and Evolutionary Considerations in the Caecilian, Typhlonectes Natans (Amphibia: Gymnophiona). Acta Zool..

